# Paving the Road for Modern Particle Therapy – What Can We Learn from the Experience Gained with Fast Neutron Therapy in Munich?

**DOI:** 10.3389/fonc.2015.00262

**Published:** 2015-11-27

**Authors:** Hanno M. Specht, Teresa Neff, Waltraud Reuschel, Franz M. Wagner, Severin Kampfer, Jan J. Wilkens, Winfried Petry, Stephanie E. Combs

**Affiliations:** ^1^Department of Radiation Oncology, Klinikum rechts der Isar, Technische Universität München, Munich, Germany; ^2^Forschungs-Neutronenquelle Heinz Maier-Leibnitz II (FRM II), Technische Universität München, Garching, Germany; ^3^Institute of Innovative Radiotherapy (iRT), Department of Radiation Science, Helmholtz Zentrum München, Oberschleißheim, Germany

**Keywords:** fast neutron therapy, fast neutrons, reactor neutrons, RBE, adenoidcystic carcinoma, high-LET radiation

## Abstract

While neutron therapy was a highly topical subject in the 70s and 80s, today there are only a few remaining facilities offering fast neutron therapy (FNT). Nevertheless, up to today more than 30,000 patients were treated with neutron therapy. For some indications like salivary gland tumors and malignant melanoma, there is clinical evidence that the addition of FNT leads to superior local control compared to photon treatment alone. FNT was available in Munich from 1985 until 2000 at the Reactor Neutron Therapy (RENT) facility. Patient treatment continued at the new research reactor FRM II in 2007 under improved treatment conditions, and today it can still be offered to selected patients as an individual treatment option. As there is a growing interest in high-linear energy transfer (LET) therapy with new hadron therapy centers emerging around the globe, the clinical data generated by neutron therapy might help to develop biologically driven treatment planning algorithms. Also FNT might experience its resurgence as a combinational partner of modern immunotherapies.

## Introduction

Radiation therapy is one of the three essential pillars of cancer treatment. Today photon treatment delivered by linear accelerators is the most commonly used treatment modality. There is, however, a strong physical and biological rationale for the use of particle therapy in radiation oncology. Due to the high relative biological effectiveness (RBE), neutron beam therapy might offer an advantage compared to photon beam therapy, especially in the treatment of malignancies known to be radioresistant (1, 2). This is a result of the high linear energy transfer (LET), which is in the range of about 200 keV/μm for 2 MeV neutron beams, about 200-fold higher than with conventional photon beams (3). The RBE for a 2 MeV neutron beam is estimated to be somewhere between 2 and 7 (4). This means that 1 Gy delivered by fast neutron therapy (FNT) should be as effective in killing cancer cells as 2–7 Gy of photon treatment. The numbers stated here implicate large uncertainties with the RBE varying between different tumor entities and even within the entities depending on the tumor grading (5). Especially for brain tumors and late reacting tissues, the RBE is estimated to be in the upper part of the range (6).

Neutron therapy might be able to overcome the negative effect of tumor hypoxia, since the oxygen enhancement ratio of neutrons is only about 1.3 compared to up to 3 in photons (7). Furthermore, there is only a weak dependency on the cell cycle, meaning that non-proliferating cells can also be effectively targeted with neutron therapy (8).

International clinical trials were enthusiastically embraced from the mid 1970s through the mid 1980s, only to be abandoned in the late 1980s as clinicians observed unacceptable side effects. Although the number of patients treated with FNT up to today might be as high as 30,000, so far, large randomized studies comparing neutron therapy to standard photon radiation are not available and they will probably not be carried out in the future. For certain indications studies in the past clearly indicated a favorable outcome in terms of local control (LC) for neutron treatment compared to conventional photon treatment alone. This was shown for salivary gland tumors (9, 10), adenoidcystic carcinoma (ACC) of the trachea (11), prostate cancer (12, 13), pleural mesothelioma (14), or malignant melanomas (15, 16). Although most of these studies only recruited small patient numbers, for certain indications such as incompletely excised or unresectable salivary gland tumors, neutron therapy not only achieved superior LC (17) but also improved overall survival (OS) (18). To further increase the efficacy of neutron therapy and to reduce unwanted side effects, efforts were made to improve treatment conformality by introducing 3-D treatment planning systems (19–21). The Karmanos Cancer Center FNT facility in Detroit, MI, USA even had a delivery system for intensity modulated radiotherapy (IMRT) commissioned, but it was shut down in 2011. Up to today, four FNT facilities continue to operate worldwide as depicted in Table 1 (22).

**Table 1:** **Current status of operating neutron facilities worldwide [status as stated at the IAEA Technical Meeting 2013 (F1-TM-44771)]**.

Location	Source	Mean energy (MeV)	50% depth (cm)	Beam direction	Collimator type	First patient treated	Patients treated (*n*)
University of Washington Medical Center, Seattle, USA	Cyclotron p(50.5) + Be	20	14	Isocentric	Multi leaf	1984	2960
iThemba Laboratory for Accelerator Based Science (LABS), Cape Town, South Africa	Cyclotron p(66) + Be	25	16	Isocentric	Multi blade trimmer	1988	1788
Tomsk Polytechnic University, Tomsk, Russian Federation	Cyclotron d(13.6) + Be	6.3	6	Horizontal	Inserts	1983	1500
FRM II, Technische Universität München, Garching, Germany	Uranium converter	1.9	5	Horizontal	Multi leaf	2007	124

Besides FNT, where mean neutron energies range between one and a few tens of MeV, neutron therapy can also be delivered as boron neutron capture therapy (BNCT). For this second branch of neutron therapy, low energy neutrons [thermal (<0.5 eV) or epithermal neutrons (0.5–10 keV)] but high neutron fluxes are needed (as they can be delivered by research reactors) and boron compounds are injected in order to selectively damage tumor cells. However, because patient treatment with BNCT was never carried out at the research reactor in Munich, results observed with BNCT will not be covered here and the difficulties regarding the compound biological effect (23), and the physical dose calculation will not be further discussed in this article.

This article aims to describe the experience gained with FNT in Munich within almost 30 years of clinical use and to outline how this experience might be of clinical relevance in modern particle therapy.

## Materials and Methods

### Neutron Therapy in Munich Between 1985 and 2000 (FRM I)

**Figure 1:**
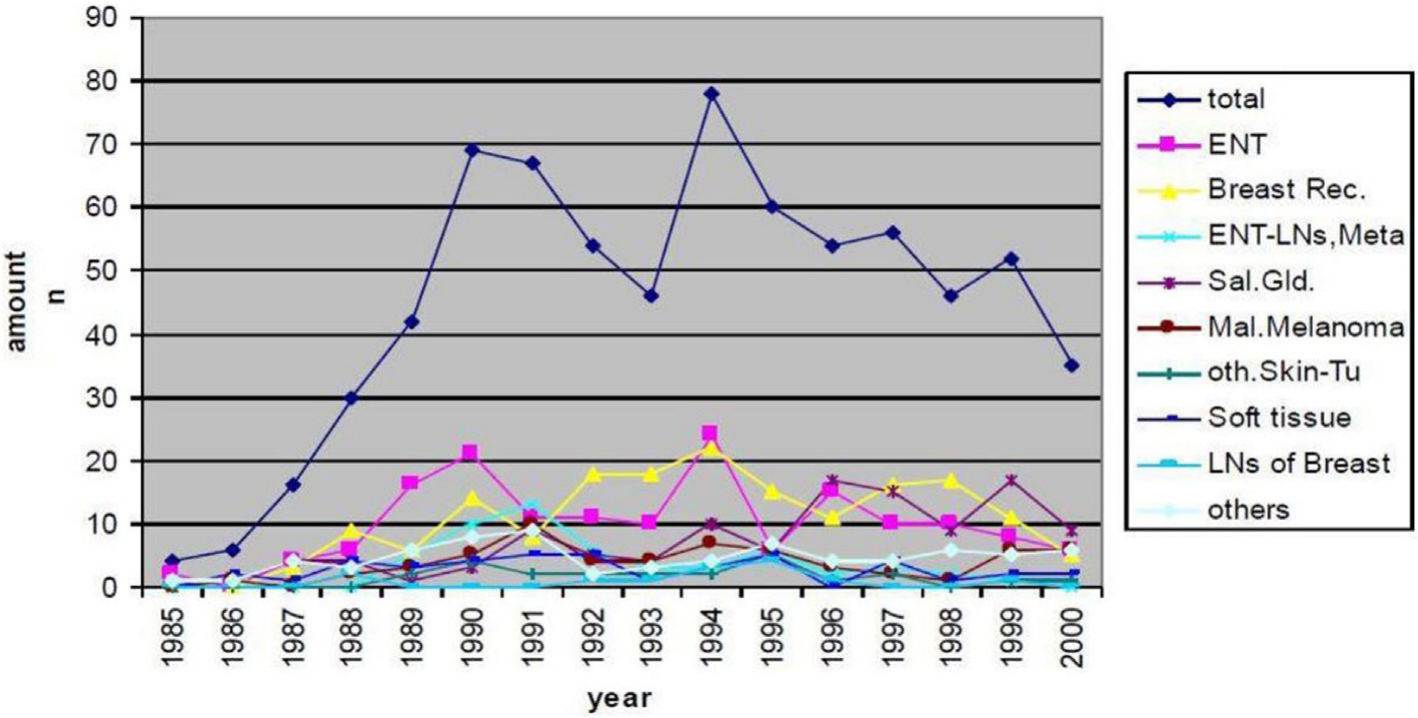
**Patient numbers and treatment indications of FNT at FRM I (RENT facility) between 1985 and 2000**.

Neutron treatment in Munich started at the first research reactor in the so called Reactor Neutron Therapy (RENT)-facility. From 1985 until the shutdown of the reactor in 2000, 715 patients were treated with FNT. Treatment indications and patient numbers are depicted in Figure 1. The main treatment indications were curative treatment of salivary gland tumors, curative and palliative treatment of head-and-neck cancers and palliative treatment of breast cancer recurrences. Over the years the indication spectrum changed; while earlier patients were treated in curative intent for salivary gland tumors or other lesions, recently, treatment has shifted to palliative indications, predominantly skin metastases mainly from breast cancer or malignant melanoma. Most of the patients were treated in combination with conventional photon or electron beams, where FNT was applied as a local boost. Most commonly, 3–5 fractions at a single dose of 2 Gy (physical dose, RBE approximately 3) were applied to the center of the tumor (24).

Of these 715 patients, 48 patients with ACC of the salivary glands were evaluated for LC and OS as well as for treatment related toxicities. Patients were at a median age of 55 years, and most patients had received surgery prior to FNT. After conventional photon irradiation with 2 Gy single dose up to a median total dose of 50 Gy (range 50–56 Gy) a median neutron dose of 6 Gy (range 4.5–7.5 Gy) at a median single dose of 1.5 Gy (range 1.5 Gy–2 Gy) was applied. Patient characteristics in detail are shown in Table 2.

**Table 2:** **Characteristics of patients with adenoidcystic carcinomas of the salivary glands treated with FNT**.

	n
Age (years)	55 (17–80)
Prior therapy	
Surgery	45 (94%)
Biopsy	3 (6%)
Resection-status after surgery	
R0	12 (25%)
R1/2	33 (69%)
Pathological Tumor Stage after surgery	
pT1/2	24 (44%)
pT3/4	18 (38%)
Lymphatic spread	10 (21%)

Moreover, 46 breast cancer patients with local recurrences on the thoracic wall were evaluated for initial treatment tumor response within the macroscopic tumor and for LC. Median time to ipsilateral chest wall recurrence after initial cancer treatment was 22 months (range 4–65 months). If the time interval between conventional photon RT within primary treatment and chest wall recurrence was shorter than 12 months, patients were treated with FNT only at a total dose of 10 Gy and a single dose of 2 Gy (1–2 times a week). If the time interval between initial treatment and cancer recurrence was more than 12 months, first re-irradiation with conventional, normo-fractionated photon radiotherapy was applied to the area of recurrence up to a total dose of 30 Gy (2 Gy daily). Afterwards macroscopic tumor lesions received a neutron boost with a single dose of 2 Gy up to a total dose of 6 Gy (1–2 times a week).

### New Research Reactor – Improved Treatment Conditions at FRM II

**Figure 2:**
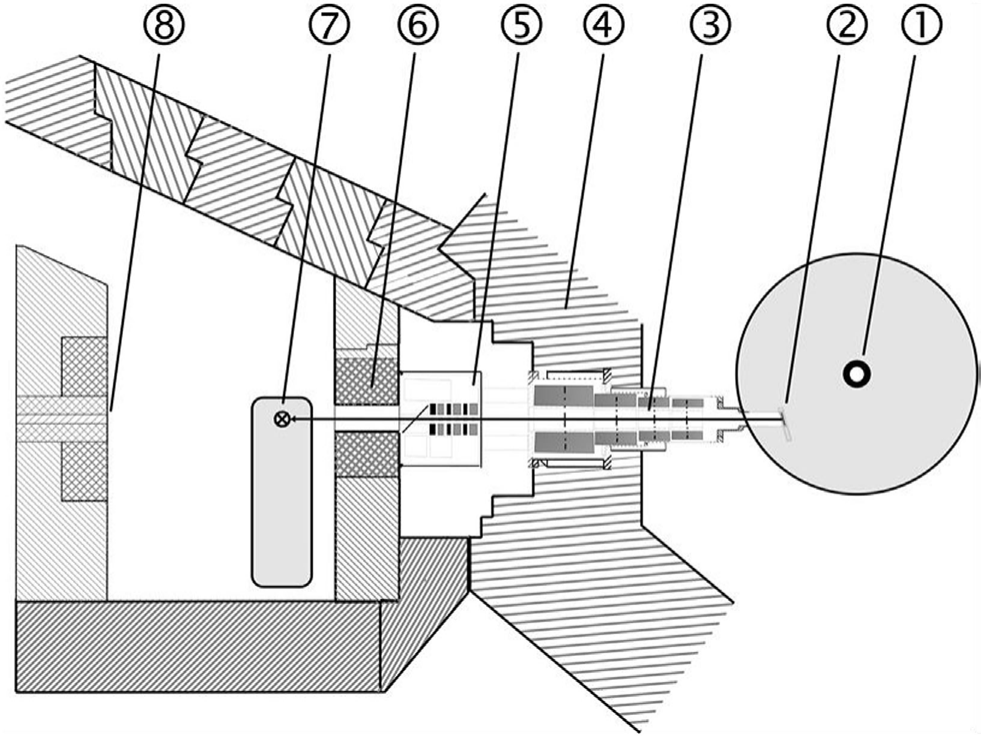
**Layout of the FNT facility at the new research reactor (FRM II): 1 Reactor core, 2 Neutron converter, 3 Neutron beam line, 4 Reactor pool wall, 5 Filter unit, 6 Multi leaf collimator, 7 Treatment table, 8 Beam stopper**.

Treatment at the new research reactor Forschungs-Neutronenquelle Heinz Maier-Leibnitz (FRM II) started in 2007 under improved conditions. The most noticeable change is the installation of a multi leaf collimator (MLC) with a maximum field size of 30 cm^2^ × 20 cm^2^. Like RENT, the beam is characterized by a neutron–photon mix and applied at a dose rate of 0.52 Gy/min neutron dose and 0.20 Gy/min photon dose in a depth of 2 cm (25). Fast neutrons are generated by a nuclear fission reactor and a uranium converter plate. The patients can be positioned in front of the horizontal beam line by a 3-D motorized couch (Figure 2).

**Figure 3:**
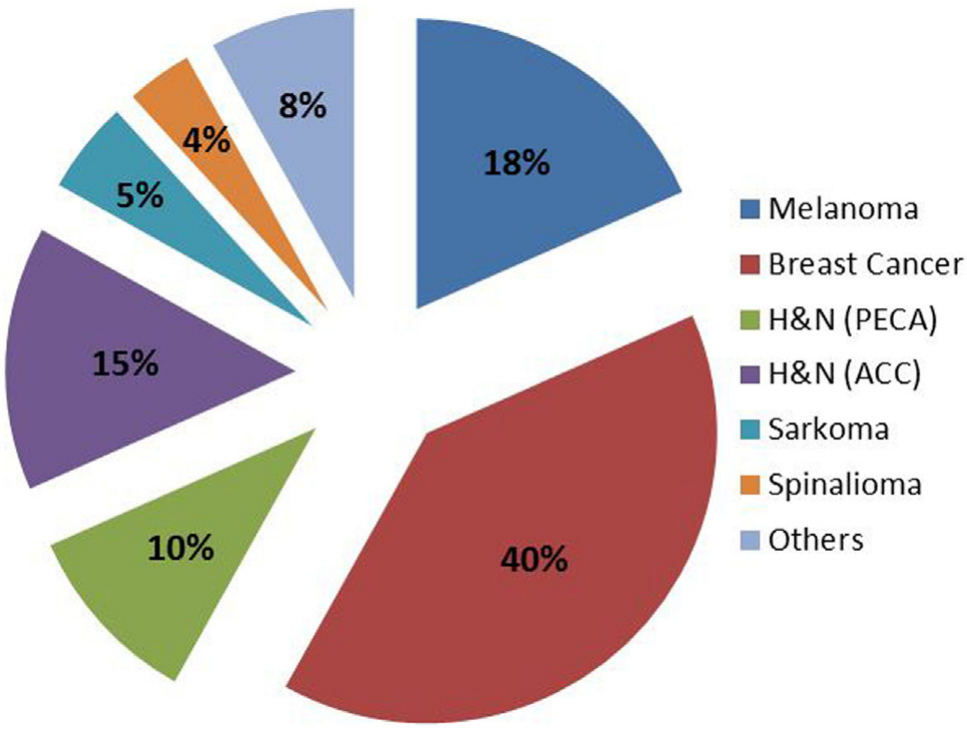
**Primary tumors of patients treated with FNT between 2007 and 2013 at FRM II**.

**Figure 4:**
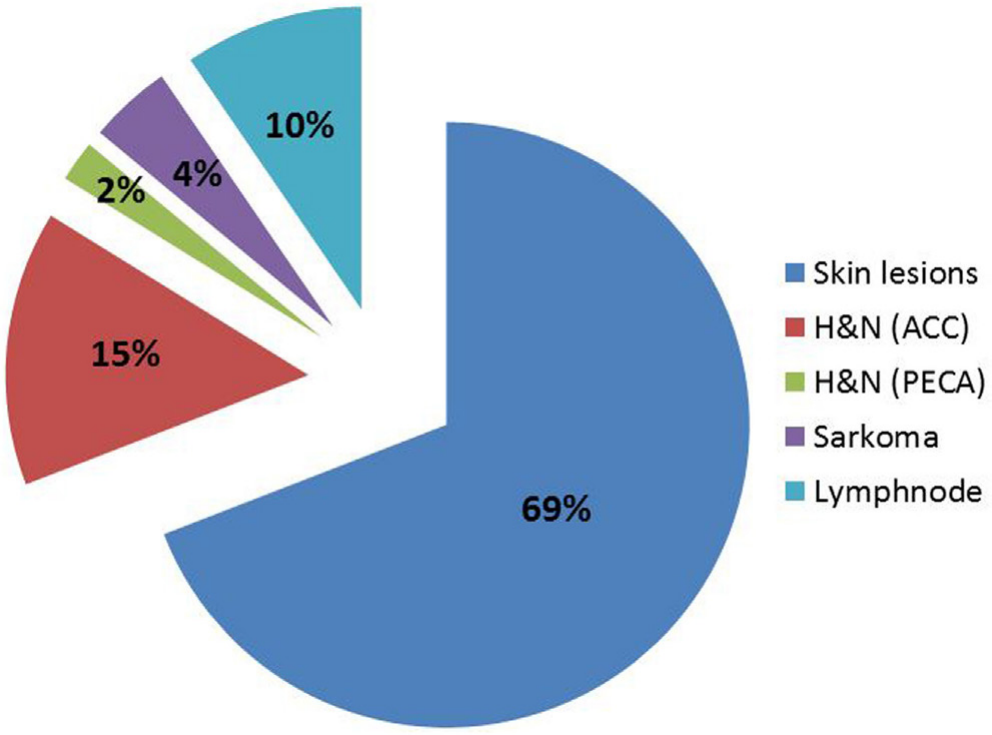
**Treatment indications for FNT at FRM II between 2007 and 2013**.

Between 2007 and 2013, 124 patients were treated, until the reactor was shut down due to major revisions. FNT continues since July 2015. Again, for most patients FNT was used as a local boost following external photon therapy. Patients treated at FRM II were between 19 and 94 years old (median 64) and the main primaries were breast cancer (40%), malignant melanoma (18%), and head-and-neck cancers (squamous 10%, ACC 15%, Figure 3). Most of the treatment indications were superficial skin lesions (69%) followed by salivary gland tumors (15%) and lymph node metastases (10%, Figure 4). A median total dose of 6 Gy (max 12 Gy, min 1.4 Gy) at a median single dose of 2 Gy was applied. For head-and-neck cancer patients thermoplastic mask systems were used and Computer tomography (CT) imaging acquired in treatment position was used to define the target volume and optimal beam positioning. For superficial skin lesion, the target volumes were determined clinically and light fields were used to define the optimal treatment position and MLC shape.

Thirty seven patients with superficial skin lesions were evaluated for tumor response and the effect of FNT on their quality of life (QoL) was also evaluated. A median total dose of 6 Gy (range 2–12 Gy) was applied at a median single dose of 2 Gy. Mean treatment field size was 12.6 (±6.6 cm) × 10.2 cm (±4.2 cm), and FNT was applied with a mean treatment time of 162 s (±23.5 s) per session.

## Ethics Statement

All patients were treated in accordance with the principles of the Declaration of Helsinki. The scientific use of retrospective data has been explicitly allowed by Bavarian federal law (BayKrG Art 27(4)). Additionally, all patients gave their written informed consent and agreed that their scientific data could be used.

## Results

Due to the manifold treatment indications that were treated with FNT between 1985 and 2013, we focused on subgroup of patients for data presentation in the present manuscript in order to deliver data comparable to with other treatment possibilities. Therefore groups of patients were pooled according to the entity treated and the technique used and analyzed separately with special focus on LC, OS, and toxicity. Because of the relatively low numbers of patients treated with FNT compared to conventional photon therapy and due to the individual character of FNT treatments, a thorough patient follow up was enforced. Patients were regularly seen in a dedicated outpatient department and if patients did not show up for their appointment, their general practitioners were contacted for follow up and toxicity information.

### Primary Treatment for Adenoidcystic Carcinomas of the Salivary Gland at RENT

Patient data was analyzed at a median follow up time of 8 years (range 2–17 years). In terms of LC, only 15% of the patients showed local tumor progression within the initial site during follow-up. This resulted in LC rates at 5, 10, and 15 years of 90, 85, and 85%. Eighty percent of the patients were still alive after 5 years. This dropped to 60% after 10 years due to the development of distant metastases, as they are common in this tumor entity. Five patients (10%) showed severe late side effects in terms of skin toxicity with ulceration of the skin and seven patients (15%) developed osteonecrosis within the mandible.

### Palliative Treatment for Thoracic Wall Recurrences at RENT

Patient data were analyzed at a median follow up time of 23 months (range 4–65 months). More than 2/3 of the patients (68%) showed complete remission of macroscopic chest wall metastases within the radiation field during follow up. Another 29% of the patients at least showed partial remission leaving only one patient without tumor response after FNT. Median time to tumor progression (in- and outside of the treatment field) was 9 month and LC after 3 years was 55%. After FNT (with or without photon treatment) patients showed acute side effects with radiodermatitis up to grade II (CTCAE V4.0). During follow-up, five patients (10%) showed a grade II fibrosis within the treatment field, no grade III or IV side effects were observed.

### Palliative Treatment for Metastatic Skin Lesions at FRM II

In terms of tumor response macroscopic skin lesions showed a good response after FNT with 25% achieving complete remission, 56% partial remission and 19% had stable disease within the treated region at first follow up 6 weeks after completion of FNT. Nearly all patients (97%) stated that their personal situation was improved by the FNT.

## Discussion

**Figure 5:**
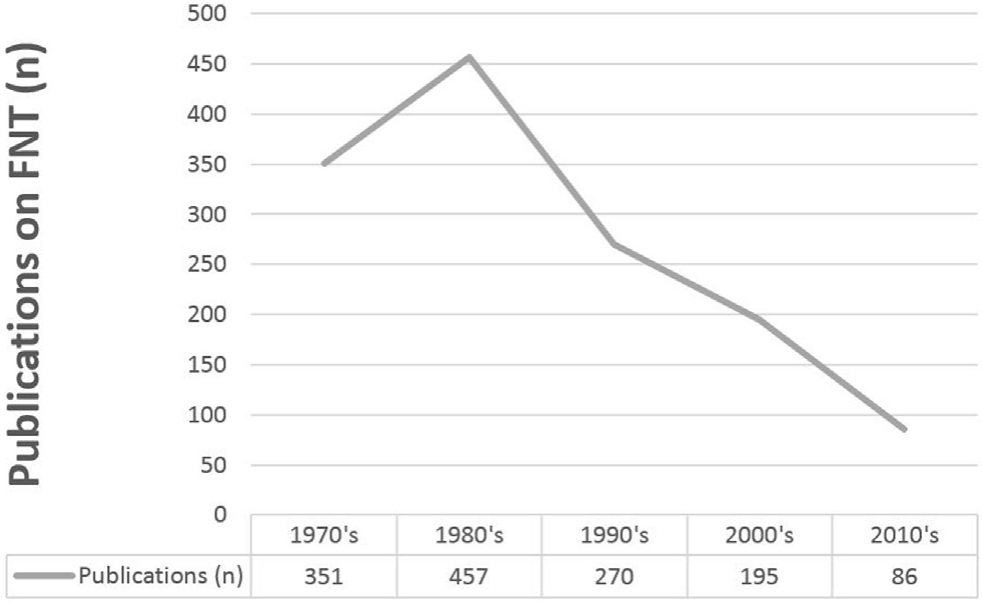
**Manuscripts on FNT (PubMed search: Fast neutron therapy, results pooled by decades)**.

Although FNT has been around since the 1970s, the number of publications on this topic is limited and unlike in most fields of oncology, the number of publications per year has not increased, but even decreased over the last decades (see Figure 5). This is explained by the clinical difficulties that developed during the application of FNT: In spite of benefits based on the biological properties of neutrons, the rates of treatment-induced side effects limited the widespread use of neutron therapy, and thus almost removed neutron centers from clinical radiation oncology (9, 26, 27). For certain indications, however, few centers are still in operation, including also approaches with BNCT (28–30).

Due to the distinct physical features of neutrons, radiation protection remains challenging and high precision modern radiotherapy, as it can be delivered by a modern linear accelerator with photons, requires tremendous efforts in terms of costs and infrastructure to realize. Horrible side effects, which were caused in the early days of FNT when the new technique was enthusiastically embraced and the knowledge on dosing and treatment planning was not yet existent, sank deep into the collective memory (31–35). Also the clinical use of a fixed neutron RBE was bound to cause problems, despite good radiobiological data which showed its variability. There are several papers describing the theoretical difficulties associated with neutron RBE’s and how difficult it would be to improve the therapeutic ratio by using biological effective dose comparisons and modeling the relation between RBE and dose per fraction (36, 37). Therefore it seems like the radiation community is about to draw the veil of oblivion over FNT.

Even if the knowledge on FNT is still limited and the studies that were carried out so far will not hold up to today’s standard of randomized, well controlled studies and even if there are some authors who question an advantage of High-LET radiation in terms of tumor control at all (38) it can be stated that there is conclusive evidence on the capability of FNT to offer improved LC compared to photon treatment (9, 29, 39, 40). And this is also true for tumor entities that are known to be resistant to conventional radiation treatment. However, even if LC can be achieved, this often does not lead to a favorable OS, since survival is often limited by early distant metastases, as it was just recently shown by Liao et al. (16) and as it is also supported by our data on ACC patients.

Therefore in most treatment indications, FNT is considered useful today, and OS might not be the appropriate tool to measure the treatment success. We saw some tremendous improvement in QoL for individual patients due to tumor regression and reduced effort in wound care. Most of the patients treated with FNT stated that they had profited from this treatment and that their personal situation was improved after FNT. The main reason for this is, as stated above, in our opinion the good tumor response. Since most lesions treated were superficial, the treatment success was also easily comprehensible to the patient. So it can be stated that for superficial metastases, FNT offers a well-tolerated and effective treatment option. The high RBE also leads to short treatment times and few treatment sessions, which adds to the attractiveness in terms of a palliative treatment option.

The experience made with FNT should be evaluated carefully since it might be useful not only to learn more about FNT itself, but also about high-LET particle therapy in general. Since neutron therapy has been around since the 1970s, there are long-term survivors that can help to identify risk and chances associated with heavy-particle therapy. It has to be mentioned in this regard that there is a potentially increased risk of secondary malignancies caused by neutron irradiation, especially to healthy tissues outside of the treatment field. Large concerted efforts such as the Euratom Allegro project are currently carried out to further evaluate this topic (41). In the treatment of patients with ACC of the salivary gland, we saw some patients with osteonecrosis of the mandibular bone. Of course the relative numbers of those complications are considerably high compared to the numbers we are accepting in the photon community. But it also has to be considered that some of these patients had large tumors that were already infiltrating the bone, so the reason for the osteonecrosis is not necessarily the treatment but the tumor itself. This fact is reflected by the high rate of incomplete resections (more than 2/3 of the evaluated patients), and it can certainly be seen as a selection bias against FNT. But still, this is also a warning that high LET-radiation and comparatively high single doses might not be appropriate in sensitive areas, especially when the tools to determine the anatomical dose application are limited.

Another point, why it might be too early to forget about FNT, is the recent developments in cancer immunotherapy. In 2013, immunotherapy was elected as the breakthrough of the year (42). Now, even in some patients with metastasized melanoma, long-term survivors can be found (43). The combination of radiotherapy and immunotherapy seems to be a fruitful collaboration. Since immunotherapy is able to offer improved OS but often fails to achieve LC in progressive tumor lesions, local radiotherapy might prove to be an ideal combination. Not only can radiotherapy lead to LC within the treatment field, it can even cause tumor regression outside of the treatment field, the so called abscopal effects (44). So far neither the optimal time point to combine the modalities nor the optimal dosing schedule is known. But there is reason to believe if the radiotherapy would cause a greater immune-stimulatory effect, than the collaboration between the two treatment partners would be even more effective. FNT might be able to achieve this immune-stimulatory effect due to its high LET. Since FNT is usually applied only once or twice per week, lymphocyte depletion within the treatment field, as it is caused by daily routine radiotherapy, might be less pronounced. Thus, especially in patients were immunotherapy is appropriate, such as malignant melanoma, combination with FNT for skin lesions could be a promising alternative.

## Conclusion

Fast neutron therapy using reactor generated fission neutrons is limited due to the relatively low penetration depth of the beam. Therefore, in most cases, therapy is limited to superficial lesions as they often occur in recurrent breast cancer at the thoracic wall or in recurrent malignant melanoma (skin lesions). To measure the treatment success in these palliative concepts, determination of the OS or progression free survival might not be an appropriate tool. Some clinically meaningful improvements in terms of local tumor regression with relatively low side effects were observed, leading to improved QoL and a reduced effort for wound management for the individual patients. Patients treated with FNT should be treated within clinical study protocols and the remaining neutron facilities should share their experiences, as it is done for other hadron therapies as well (45). As there is a growing interest in high-LET therapy with a growing number of hadron therapy centers around the globe, the clinical data generated by neutron therapy might help to develop biologically driven treatment planning algorithms. Also recent advances in immunotherapy call to reevaluate the benefit of neutron therapy, where good local tumor control can be achieved within short treatment times and immune-modulatory effects might be more pronounced compared to conventional irradiation.

## Author Contributions

Study design: HS, JW, WP, SC. Acquisition of data: HS, TN, WR. Analysis and interpretation of data: HS, SK, SC. Drafting of manuscript: HS. Initial critical revision: WR, FW, SK, JW, SC. Revision of manuscript: HS, JW, FW.

## Conflict of Interest Statement

The authors declare that the research was conducted in the absence of any commercial or financial relationships that could be construed as a potential conflict of interest.
